# 

**Published:** 1863-04

**Authors:** 


### CONTENTS.

ORIGINAL ESSAYS AND COMMUNICATIONS.

Metals and Alloys. By Dr. B. Wood........ 147

Professional Individuality. By Geo. F. Foote, M. D........ 154

PROCEEDINGS OF SOCIETIES.

Proceedings of the Dental College Association........ 156

CORRESPONDENCE.

Annual Commencment of the " Pennsylvania College of Dental Surgery"........ 158

SELECTIONS.

Dentition and its Derangements. By A. Jacobi, M. D........ 160
Nitrous Oxide in Asphyxia. By Geo. J. Zeigler, M. D........ 163
Artificial Dentures. By W. H. Atkinson, M. D........ 165
Introductory Address. By Anthony Hockley, M. C. D. E........ 170
Concerning the Opening of Abscesses. By Nullopath........ 186

EDITORIAL.

Patience and Patients........ 189
Ohio College of Dental Surgery........ 190
The Mississippi Valley Association........ 191
Cincinnati Dental Association........ 192

